# LncRNA FOXP4-AS1 Promotes Progression of Ewing Sarcoma and Is Associated With Immune Infiltrates

**DOI:** 10.3389/fonc.2021.718876

**Published:** 2021-10-26

**Authors:** Jiachao Xiong, Liang Wu, Lu Huang, Chunyang Wu, Zhiming Liu, Wenqiang Deng, Shengbiao Ma, Zhenhai Zhou, Honggui Yu, Kai Cao

**Affiliations:** ^1^ The Orthopedics Hospital, The First Affiliated Hospital of Nanchang University, Nanchang, China; ^2^ Department of Orthopedics, The Second Affiliated Hospital of Nanchang University, Nanchang, China; ^3^ Child Health Department of the Maternal and Children Health Hospital of Jiangxi Province, Nanchang, China

**Keywords:** FOXP4-AS1, Ewing sarcoma, extracellular vesicles, tumor immune microenvironment, ceRNA

## Abstract

Ewing sarcoma (ES) is a highly malignant primary bone tumor with poor prognosis. Studies have shown that abnormal expression of lncRNA influences the prognosis of tumor patients. Herein, we established that FOXP4-AS1 was up-regulated in ES and this correlated with poor prognosis. Further analysis illustrated that FOXP4-AS1 down-regulation repression growth, migration, along with invasion of ES. On the contrary, up-regulation of FOXP4-AS1 promoted the growth, migration, as well as invasion of ES. To explore the mechanism of FOXP4-AS1, Spearman correlation analysis was carried out to determine genes that were remarkably linked to FOXP4-AS1 expression. The potential functions and pathways involving FOXP4-AS1 were identified by GO analysis, Hallmark gene set enrichment analysis, GSEA, and GSVA. The subcellular fractionation results illustrated that FOXP4-AS1 was primarily located in the cytoplasm of ES cells. Then a ceRNA network of FOXP4-AS1 was constructed. Analysis of the ceRNA network and GSEA yielded two candidate mRNAs for FOXP4-AS1. Results of the combined survival analysis led us to speculate that FOXP4-AS1 may affect the expression of TMPO by sponging miR-298, thereby regulating the malignant phenotype of ES. Finally, we found that FOXP4-AS1 may modulates the tumor immune microenvironment in an extracellular vesicle-mediated manner. In summary, FOXP4-AS1 correlates with poor prognosis of ES. It promotes the growth, migration, as well as invasion of ES cells and may modulate the tumor immune microenvironment.

## Introduction

Ewing sarcoma (ES), also known as Ewing family tumors, includes Ewing sarcoma, extraosseous Ewing sarcoma (EES), PNET, chest malignant small-cell tumor (Askin sarcoma), and aberrant Ewing sarcoma (1). ES, a primary, and highly malignant bone tumor of the bone marrow that mainly occurs in children, as well as adolescents, is typified by an incidence rate of 1/1.5 million, with the highest incidence recorded in 15-year-olds (2). Under the microscope, ES appears as small round cells with deeply stained nuclei, inconspicuous nucleoli, sparse cytoplasm, unclear cell boundaries, and occasional mitotic images (3). Although ES can occur in any part of the body, it is most dominant in the pelvis and the proximal long bones. The disease is highly malignant, and is further characterized by a short course, and rapid metastasis (4), with approximately 30% of patients manifesting apparent metastases at diagnosis. Metastasis predominantly occurs in the lungs, bones, and the bone marrow (5). ES has dismal prognosis, exhibiting a five-year survival rate of 70-80%, which worsens to 30% in patients with metastatic tumors (6–8). Therefore, understanding occurrence and the underlying mechanism of ES development will aid in identification of novel biomarkers for development of early diagnostic and treatment therapies, thereby improving and reducing survival and recurrence rates, respectively.

Advancements in transcriptome sequencing technology have enabled elucidation of the vital role played by long non-coding RNA (LncRNA) in normal development and physiological functions of cells (9, 10). Notably, impaired LncRNA expression has been implicated in transformation of malignant tumors, including maintaining cell growth, proliferation, avoiding growth inhibitors, ensuring continuous replication, promoting metastasis and invasion, as well as inducing angiogenesis, inhibiting cell apoptosis, sugar and fat metabolism, among others (11–13). Previous researches have also identified some tumor-specific lncRNAs that are tightly linked to the occurrence, development, and prognosis of tumor. These have potential as prognostic molecular markers for tumors and may provide targets for future development of diagnostic and treatment strategies (14, 15).

Previous researches have documented that FOXP4-AS1 is upregulated in various tumors, where it enhances tumor growth, migration, invasion, and other processes, and is further linked to poor prognosis of many tumors. For example, FOXP4-AS1 was upregulated in colorectal cancer (CRC), with its inhibition found to suppress cell growth and induce apoptosis both *in vivo* and *in vitro*. Moreover, FOXP4-AS1 was found to be a poor predictive factor for individuals with colorectal cancer (16). Besides, another research documented that FOXP4-AS1 was upregulated in prostate cancer (PCa), while it reportedly upregulated FOXP4 expression *via* sponging miR-3184-5p, contributing to the occurrence and progression of PCa (17).

Herein, we explored the function of FOXP4-AS1 in Ewing sarcoma *via* bioinformatics analysis and *in vitro* assays. Our data illustrated that FOXP4-AS1 is highly expressed in Ewing sarcoma tissues and cells, with this phenomenon linked to poor prognosis of individuals with ES. Functionally, it regulates cell proliferation, migration, invasion of Ewing sarcoma and may modulates the tumor immune microenvironment in an extracellular vesicle-mediated manner. These findings provide new insights into the underlying mechanism of ES occurrence and progression, and are expected to aid in future development of diagnostic and treatment strategies.

## Materials and Methods

### Expression Analysis and Data Processing

We analyzed expression data for FOXP4-AS1 in pan-cancer patients, obtained from The Cancer Genome Atlas (TCGA), as well as Genotype-Tissue Expression (GTEx) database. These datasets were downloaded from the UCSC Xena TOIL project (18, 19) and the Transcript per Million (TPM) data has been log-transformed. All microarray data were abstracted from the Gene Expression Omnibus (GEO) database, and a summary is presented in **Table S1**. Batch effects were corrected *via* the Limma package (v3.13) function “removeBatchEffect” (20) and all microarray data has been log-transformed. R packages ‘tidyr’ (v1.1.3), ‘dplyr’ (v1.0.7), ‘tibble’ (v3.1.3) were used for data cleaning. All data processing was implemented in Rstudio software version 4.0.3.

### Cell Lines and Culture Conditions

Human ES cell lines A673 and SK-N-MC are persevered in our laboratory. A673 cells were cultured in high-glucose DMEM medium, while SK-N-MC cells were maintained in Minimum Essential Medium (MEM). Human bone marrow-derived mesenchymal stem cells (BM-MSCs) and media (BM-MSCs growth medium) were purchased from Shanghai Zhong Qiao Xin Zhou Biotechnology Co., Ltd. Both media were enriched with 10% FBS, 100 Units/mL penicillin, along with 100 μg/mL streptomycin, and cell cultures maintained in humidified air at 37°C with 5% CO_2_.

### Cell Transfection

To knock down FOXP4-AS1, we designed small interfering RNAs (siRNAs) targeting FOXP4-AS1 and a corresponding negative control and synthesized them at the Shanghai HanHeng Biotechnology Co., Ltd (**Table S2**). We also purchased a FOXP4-AS1 overexpression plasmid (pcDNA3.1-FOXP4-AS1) from Shanghai HanHeng Biotechnology Co., Ltd for overexpression assays. Cells were transfected for 48 h, using Lipofectamine^®^ 3000 transfection reagent (Cat No, Invitrogen, Thermo Fisher Scientific, Inc.) as described by the manufacturer, then harvested for subsequent experiments.

### RNA Extraction From Cell Lines

TRIzol reagent (Cat No, Invitrogen, Carlsbad, CA, USA) was employed to isolate RNA from ES cell lines. After purification, RNA was eluted with 20 μL of nuclease-free water, then its quantity and quality were checked using a NanoDrop2000 spectrophotometer (Thermo Scientific, Waltham, MA, USA).

### RNA Expression Analysis by Quantitative Real-Time PCR

The cDNA was generated from 2 μg of the RNA with a reverse transcription kit (Cat No, Takara, Dalian, China). Quantitative real-time PCR (qRT-PCR) was performed on an ABI 7900HT system (Applied Biosystems, CA, USA) and SYBR Green assays (PerfectStart^®^ Green qPCR SuperMix, TransGen Biotech Co., Ltd., Beijing, China), targeting FOXP4-AS1, GAPDH, U6 genes. Primers are presented in **Table S3**. The 2^-ΔΔ^CT approach was employed to determine lncRNA expression level, with GAPDH serving as the normalization standard. For subcellular fractionation assay, GAPDH and U6 were used as markers of the cytoplasm and nucleus, respectively.

### Cell Proliferation Assays

1×10^4^ cells/well were inoculated in 96-well plates (in triplicates) at 37°C and a 5% CO_2_ humidified atmosphere, then a CCK8 assay performed after 24, 48, 72 and 96 hours, as described by the manufacturer. In each group, five independent experiments were conducted, and a graph plotted, per the average value.

### Cell Migration and Invasion Assays

Cell migration along with invasion were performed in Transwell insert chambers (8 µm pores; Guangzhou Jet Bio-Filtration, Co., Ltd) and BD BioCoat Matrigel Invasion Chamber (BD Biosciences, Bedford, MA), respectively, using FBS as the chemoattractant. Briefly, we harvested the cells and resu-spended them in serum-free medium following the transfection, then approximately 1 x 10^5^ (migration assay) or 1.5 x 10^5^ (invasion assay) prepared cells introduced into the chamber, followed by a 24-h incubation at 37°C. The cells that migrated or infiltrated through the membrane were fixed (in 20% methanol), stained (in 0.1% crystal violet, Invitrogen), then imaged and counted under the microscope. The rate of cell migration along with infiltration was defined as the ratio of migrated cells in the indicated sample to that in the control sample.

### Correlation Analysis

For correlation analysis of FOXP4-AS1, Spearman’s correlation was performed on TPM data of Ewing sarcoma cell lines (CCLE database) and microarray data (GEO database) of Ewing sarcoma cells. Positively or negatively significant correlated genes were identified at a cut-off criterion of r > 0.5, p < 0.05 and r < -0.5, p < 0.05 respectively.

### Functional and Pathway Enrichment Analyses

Gene Ontology (GO) terms and enrichment analysis for Hallmark gene sets were performed to identify functional and pathway classes using Metascape (http://metascape.org) (21). Statistical thresholds were set at P<0.01, a minimum count of 3, and an enrichment factor >1.5.

### Construction of a Protein-Protein Interaction Network

A PPI network for proteins identified across BioGrid, InWeb_IM, as well as OmniPath data resources was constructed in Metascape (21, 22). The Molecular Complex Detection (MCODE) algorithm was employed to determine the densely connected network components (23).

### Gene Set Enrichment Analysis

GSEA (Gene Set Enrichment Analysis) (24) was performed on the correlation-ranked gene list of FOXP4-AS1 from ES cell lines microarray data (GSE70826, GSE17618), and remarkably enriched gene sets were identified at a cut-off criterion of p-value < 0.05. Upregulated or downregulated gene sets between MSCs and ES were then identified by the GSVA (Gene Set Variation Analysis) (25) in the GSE17674 dataset, while Hallmark gene sets were abstracted from the MSigDB (Molecular Signature Database) of Broad Institute (https://www.gsea-msigdb.org/gsea/index.jsp). Remarkably differential enriched gene sets were identified at a cut-off criterion of p-value < 0.05. The intersection between GSEA and GSVA was shown by the Venn diagram generated using the VennDiagram package in R.

### Subcellular Fractionation Assay

Approximately 1x10^7^ target cells were harvested and rinsed once with pre-cooled PBS. The supernatant was discarded then nuclear and cytoplasmic RNA extracted using the PARITM Kit (Thermo Scientific) as described by the manufacturer. Levels of FOXP4-AS1 expression, alongside GAPDH and U6 as controls, were detected using qRT-PCR as documented previously.

### RNA Immunoprecipitation Assay

RNA immunoprecipitation was performed using the Magna RIP kit (Cat No, Millipore), per the method provided by the manufacturer. Briefly, target cells with a growth confluence of more than 90% were collected, lysed using an equal volume of RIP buffer. After that, the cell lysate was inoculated overnight with magnetic beads containing the target antibody in RIP buffer, then the magnetic beads was washed three times and resuspended in RIP washing solution, and incubated with rotation. Finally, the magnetic beads were removed to obtain a supernatant containing RNA. RNA from the supernatant was isolated using TRIzol reagent, cDNA synthesized then subjected to and qRT-PCR to analyze levels of FOXP4-AS1 expression.

### Differential Gene Expression Analysis

Differential gene expression analysis was performed using the Limma R package. Down-regulated miRNAs were identified from the GSE80201 dataset, at a cut-off criterion of log2(fold change) < -0.5, p.value < 0.05. Up-regulated mRNAs were identified from the cell lines microarray data (GSE70826, GSE17618, GSE48022, GSE90970, GSE70826), at a cut-off criterion of log2(fold change) > 0.5, adjust.p.value < 0.05.

### Construction of a Competing Endogenous RNAs Network

We created a FOXP4-AS1-associated ceRNA network and visualized it in Cytoscape (22) (version 3.8.0; www.cytoscape.org). In brief, we obtained predicted target miRNAs with potential binding sites for FOXP4-AS1 through the LncBook website. Then, ceRNA-miRNAs were identified as intersecting genes across predicted target miRNAs and down-regulated miRNAs.

Furthermore, predicted target mRNAs with potential binding sites for ceRNA-miRNAs were predicted using the TargetScan (26, 27) and miRDB (28, 29) databases. After that, we incorporated the intersection between the three gene sets (predicted target mRNAs, up-regulated mRNAs, FOXP4-AS1 positively associated genes) into the ceRNA network and obtained ceRNA-mRNAs. Finally, we also combined the GSEA results to unravel the association between significantly enriched pathways and ceRNA-mRNAs.

### Analysis of Immune Infiltration and Its Correlation With FOXP4-AS1 Expression

We employed the CIBERSORT package in Rstudio (version 4.0.3) (30) to estimate the invasion of 22 immune cell kinds in ES. Differences in immune cell infiltration were explored using the Wilcoxon signed rank-sum test and compared to normal tissues at a threshold of p < 0.05. The relationship between expression levels of FOXP4-AS1 with corresponding immune-cell infiltration was examined using the Spearman rank correlation analysis.

### Extracellular Vesicles Extraction

For extracellular vesicles (EVs) purification, FBS was spun at 120,000 × g for 18 hours at 4°C to remove EVs. Then, cells were cultured in an EVs-depleted FBS medium for 48 hours, and the supernatant subjected was collected to isolate EVs by differential ultracentrifugation (UC). The supernatant was spun for 10 minutes at 300 x g to remove precipitates, at 2000 x g for 10 minutes at 4°C to remove dead cells, then at 10,000 x g for 30 minutes at 4°C to remove cell debris. Afterward, we collected the supernatant and filtered it through a 0.22 μm filter membrane, followed by 90-minute centrifugation at 100,000 x g at 4°C to allow EVs to be deposited at the bottom of the tube. We resuspended the precipitates in PBS, followed by spinning at 100,000 x g for 70 minutes at 4°C to allow EVs to be deposited at the bottom of the tube again. For RNA extraction, the EV pellet was directly lysed by TRIzol reagent. For other experiments, the EV pellet was resuspended in 100ul PBS, then stored at -80°C until subsequent manipulations.

### Nanoparticle Tracking Analysis

Particle size of the extracellular vesicles was analyzed using the NS300 (Malvern Panalytical, Malvern, United Kingdom) Nanoparticle Tracking Analysis (NTA). Briefly, the sample was diluted 100~1000 times with PBS, 1 ml of the diluted sample absorbed and manually injected into the instrument. Five 60-second videos were recorded, then a Nanosight NTA (V3.4) software used to analyze size distribution and concentration of the particles.

### Transmission Electron Microscopy

Twenty (20) μl of extracellular vesicle suspension was placed on a copper mesh grid of an electron microscope, in form of a droplet, and maintained for more than 1 minute. The droplet was fixed, for 1-10 minutes, by negative staining with a 2% uranyl acetate aqueous solution, blotted off using a filter paper, then left to airdry at room temperature. The stain was observed and photographed under a 120kv biological transmission electron microscope.

### Western Blot Analysis

Western blot analysis was carried as documented previously (4). Concisely, the RIPA buffer was employed to isolate proteins and quantified *via* the BCA assay. Equal concentrations of proteins were separated on a SDS-PAGE, then transfer-embedded onto PVD membranes (Millipore). After that, 5% non-fatty milk was employed to block the membranes, and inoculated with specified primary antibodies, namely Calnexin, HSP70, TSG101, Alix, and CD63, as described by the manufacturer. Membranes were then rinsed with the Dilute Tris Buffered Saline with Tween 20 (TBST-1x) solution, inoculated with specified peroxidase-labelled secondary antibodies and exposed with ECL kit (Millipore). All antibodies were obtained from Wuhan Sanying Biotechnology Co., Ltd (Wuhan, China).

### RNA Analysis of ES Cell-Derived EVs

For RNA expression analysis of ES cell-derived EVs, total RNA was extracted from the UC-purified EV pellet by TRIzol reagent. The quantity and quality of RNA were detected as described earlier. The expression levels of exosomal lncRNA were detected by qRT-PCR and standardized by GAPDH expression levels.

### Statistical Analyses

Statistical analyses were performed using Rstudio (v4.0.3) and GraphPad Prism 8.0. Data were given as means ± standard deviations (SD). Comparisons between two groups were conducted using an independent student’s t-test for normally distributed data. Otherwise, a Chi-square test was applied for comparisons. Individual risk ratios (HR) for overall survival time (OVS) and event-free survival time (EFS) were estimated using the Univariate Cox proportional risk regression. The r package “survival” and “survminer” were used for the survival analysis. ES patients were divided into two groups: high (≥ median value) and low (< median value) expression based on the FOXP4-AS1 expression level. The Kaplan-Meier method and log-rank test were used to estimate the relationship between FOXP4-AS1 expression and OVS and EFS. Spearman correlation test was carried out to confirm the correlation between FOXP4-AS1 and mRNA expression. All hypothesis tests were two-sided, with data followed by p<0.05 signifying statistical significance.

## Results

### FOXP4-AS1 Is Upregulated in ES Tissues and Is Linked to Poor Prognosis

**Figure 1:**
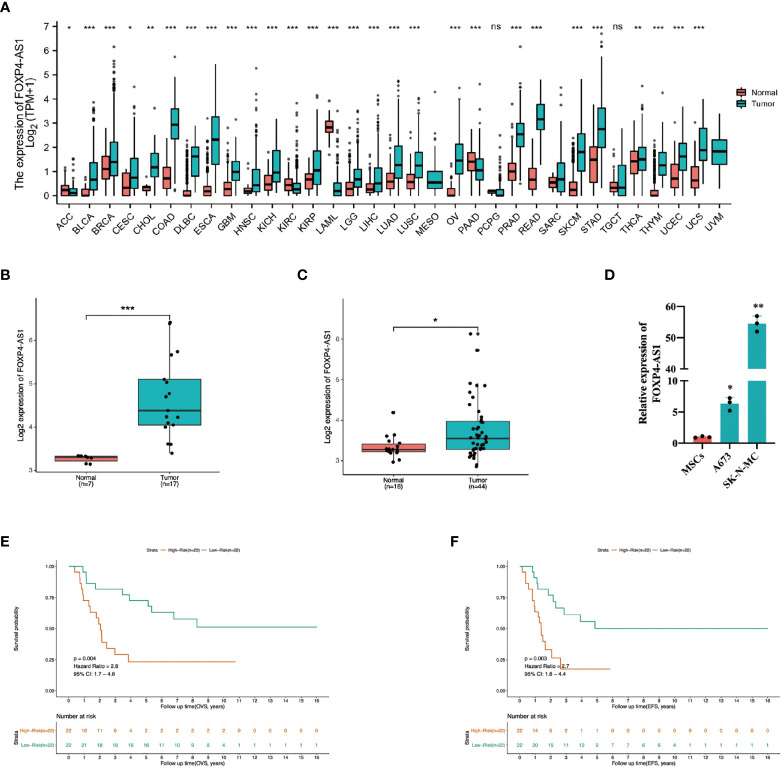
Profile of FOXP4-AS1 expression in ES and its association with poor prognosis. **(A)** Patter of FOXP4-AS1 expression in datasets from the TCGA and GTEx databases. **(B, C)** Patterns expression of FOXP4-AS1 in ES tissues and cells. **(D)** The contents of FOXP4-AS1 in ES cell lines A673 and SK-N-MC, detected *via* qRT-PCR. **(E, F)** Survival and univariate Cox regression analyses reveal the effect of FOXP4-AS1 expression on overall survival (OVS) and event-free survival (EFS) of ES patients. ^*^p < 0.05, ^**^p < 0.01, ^***^p < 0.001, ns, non-significant.

Analysis of FOXP4-AS1 expression, across 33 tumors from the TCGA and GTEx databases, revealed its upregulation in most tumors (**Figure 1A**), suggesting it potential as a poor prognostic factor in tumor patients. To date, the role of FOXP4-AS1 in ES remains unclear. Therefore, we analyzed patterns of lncRNA expression between ES and normal muscle tissues based on the GSE dataset (GSE17674). Interestingly, FOXP4-AS1 was upregulated in ES tissues (**Figure 1B**). To verify this expression, we integrated the GSE datasets (GSE48022, GSE90970, GSE70826, GSE17618) then analyzed expression of FOXP4-AS1 between ES and MSCs, and found that FOXP4-AS1 was upregulated in ES cells relative to MSCs (**Figure 1C**). qRT-PCR results corroborated these results, as evidenced by a significant FOXP4-AS1 upregulation in ES cell line A673 and SK-N-MC (**Figure 1D**). Kaplan-Meier survival curves and univariate Cox proportional analysis further proved the prognostic value of FOXP4-AS1 expression. Specifically, ES patients with high expression of FOXP4-AS1 exhibited poor overall survival (Hazard ratio = 2.8, 95% CI:1.7-4.6, p = 0.004, **Figure 1E**) and event-free survival (Hazard ratio = 2.7, Hazard ratio = 2.8, 95% CI:1.7-4.6, p = 0.004, **Figure 1F**) rates. Collectively, these data illustrate that FOXP4-AS1 might be a prospective indicator for poor prognosis in Ewing sarcoma patients.

### FOXP4-AS1 Promotes Proliferation and Mobility in Ewing Sarcoma Cells

**Figure 2:**
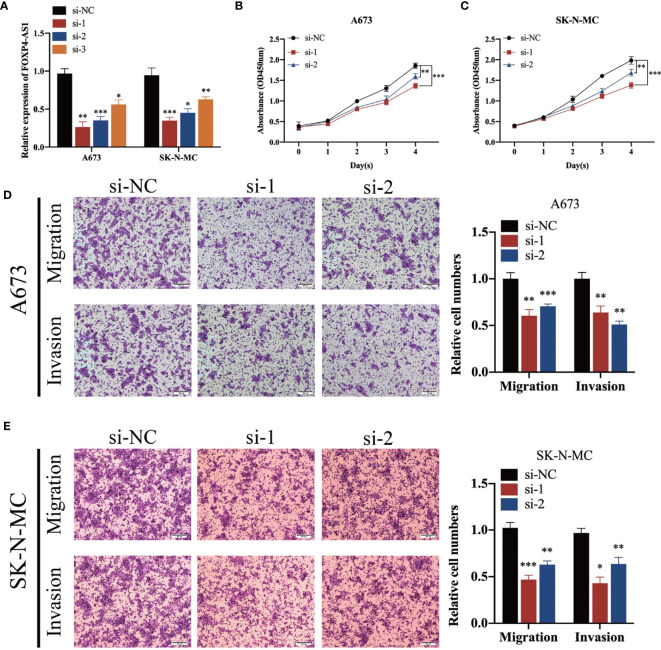
FOXP4-AS1 knockdown suppresses proliferation, migration, and invasion ability of ES cells. **(A)** FOXP4-AS1 was downregulated in ES cells after transfection with siRNA. **(B, C)** CCK-8 assay revealed that down-regulated FOXP4-AS1 suppressed the cell proliferation of ES. **(D, E)** Transwell assays revealed that knockdown of FOXP4-AS1 by siRNA significantly prevented migration and invasion of ES cells. ^*^p < 0.05, ^**^p < 0.01, ^***^p < 0.001.

**Figure 3:**
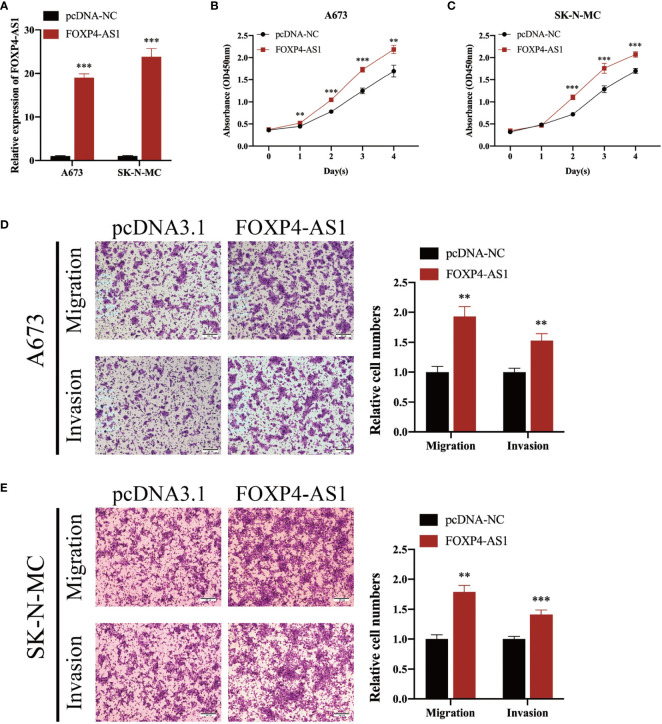
Overexpressing FOXP4-AS1 enhances cell proliferation, migration, and infiltration of ES. **(A)** Transfecting overexpression plasmid upregulated FOXP4-AS1 in ES cell lines A673 and SK-N-MC. **(B, C)** CCK-8 assay showed that FOXP4-AS1 overexpression enhanced growth of ES cells. **(D, E)** Transwell migration along with infiltration assays revealed that up-regulating FOXP4-AS1 enhanced cell migration and infiltration of ES. ^**^p < 0.01, ^***^p < 0.001.

To elucidate the biological function of FOXP4-AS1 in ES cells, we transfected short interference siRNAs (si-FOXP4-AS1) into A673 and SK-N-MC cells, to knock down FOXP4-AS1, then performed qRT-PCR to verify knockdown efficiency (**Figure 2A**). Knocking down FOXP4-AS1 remarkably suppressed growth(**Figures 2B, C**), migration, along with invasion of Ewing sarcoma cells (**Figures 2D, E**). Conversely, overexpressing FOXP4-AS1 by transfection using the pcDNA3.1 plasmid containing the FOXP4-AS1 sequence (**Figure 3A**), remarkably promoted growth (**Figures 3B, C**), migration, as well as invasion of Ewing sarcoma cells (**Figures 3D, E**). These findings indicate that FOXP4-AS1 can promote proliferation, migration, and invasion of ES cells.

### Functional Enrichment of FOXP4-AS1 Associated Genes in ES

**Figure 4:**
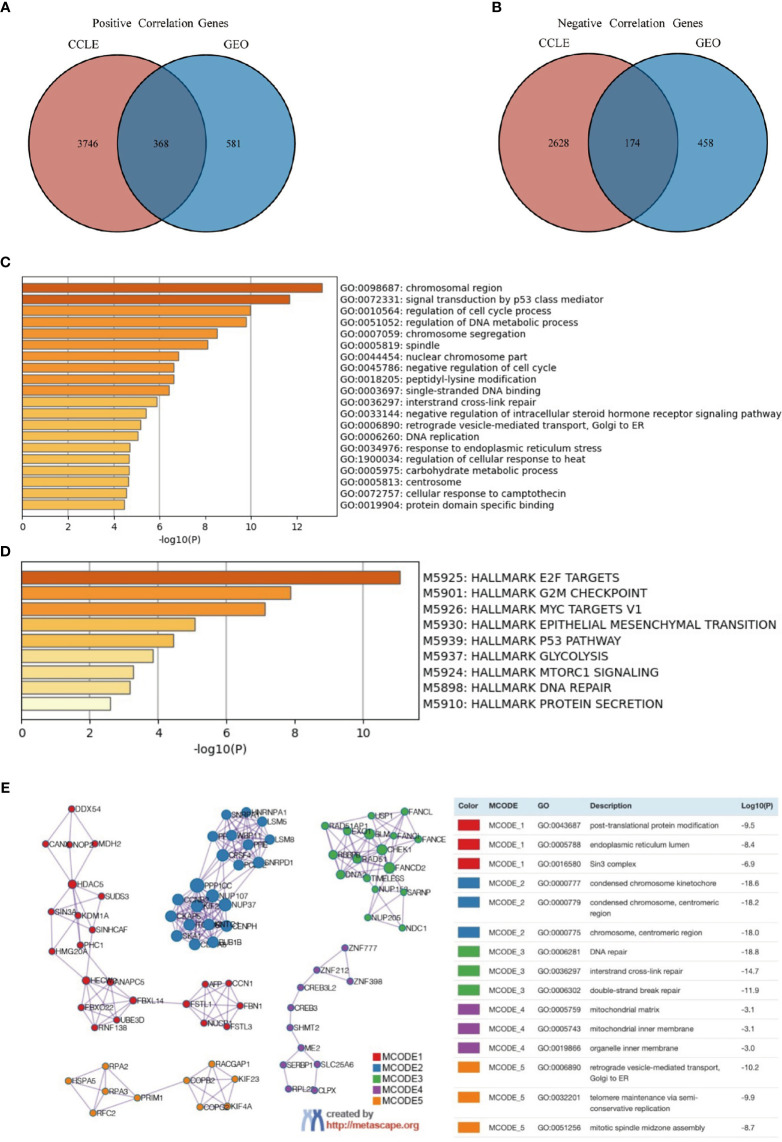
Functional enrichment of FOXP4-AS1-associated genes in ES. **(A, B)** Spearman correlation revealed that several genes that were had a significant positive correlation while others had a negative correlation with FOXP4-AS1 across datasets from both GEO and CCLE databases. The intersection of these genes is shown in the Venn diagrams. **(C, D)** Metascape was used for GO, and Hallmark Gene Sets enrichment analysis of significant correlation genes. **(E)** Metascape was used to construct a PPI network. The top five MCODE components were abstracted from the PPI network, then subjected to GO enrichment analysis.

To elucidate the functional implications of FOXP4-AS1 in ES, we analyzed enrichment patterns for FOXP4-AS1-associated mRNAs in the ES cell lines across GEO and CCLE datasets. A Venn diagram of the identified genes revealed that 368 and 174 mRNAs were positively and negatively correlated, respectively, as illustrated in **Figures 4A, B**. GO enrichment analysis illustrated that the genes were primarily enriched in chromosomal region, signal transduction *via* p53 class mediator, modulation of cell cycle process, regulation of DNA metabolic process, chromosome segregation, and spindle, among others (**Figure 4C**). On the other hand, hallmark gene sets enrichment analysis revealed various signaling cascades associated with FOXP4-AS1 expression, including E2F targets, MYC targets V1, epithelial mesenchymal transition, G2M checkpoints, p53 cascade, glycolysis, mtorc1 signaling, DNA repair, and protein secretion (**Figure 4D**). A PPI network constructed showed a significant correlation between the 542 identified genes with FOXP4-AS1 and ES. A summary of genetic constituents in the PPI network, as well as MCODE is outlined in (**Figure 4E**). The top five MCODE components were abstracted from the PPI network, then subjected to GO enrichment analysis. The top 3 MCODE components primarily included post-translational protein modification, endoplasmic reticulum lumen, Sin3 complex, condensed chromosome kinetochore, interstrand cross-link repair, centromeric region, condensed chromosome, centromeric region, chromosome, DNA repair, and double-strand break repair.

### FOXP4-AS1-Related Signaling Cascades Based on GSEA and GSVA

**Figure 5:**
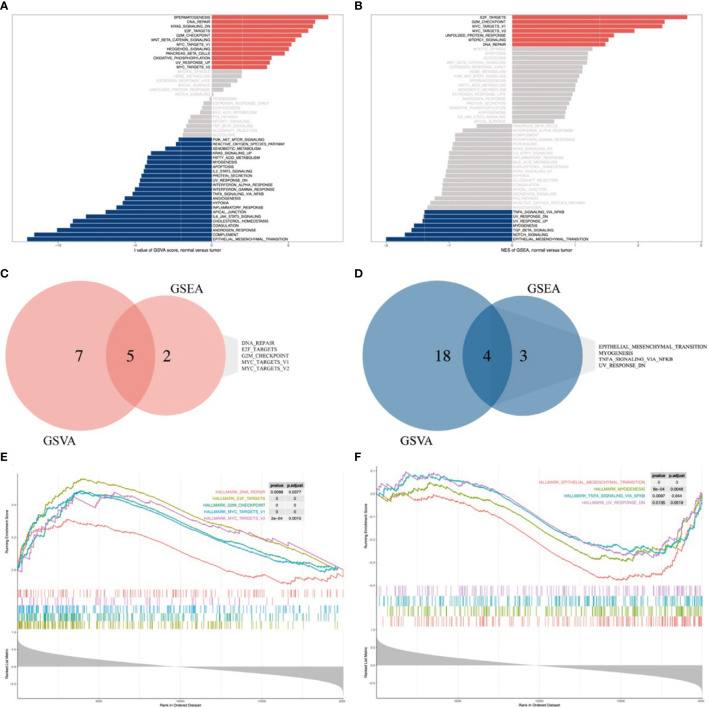
FOXP4-AS1-related signaling cascades based on genes identified from GSEA and GSVA databases. **(A, B)** Bar graphs showing results of GSVA and GSEA, respectively. All cascades, except gray, were significantly enriched (p < 0.05). **(C, E)** A Venn diagram and GSEA plot showing the commonly up-regulated cascades across GSVA and GSEA datasets. **(D, F)** A Venn diagram and GSEA plot showing the commonly down-regulated cascades across GSVA and GSEA datasets.

GSVA revealed significant differential gene sets between ES and MSCs (**Figure 5A**), and GSEA of FOXP4-AS1 correlation genes revealed significant enriched gene sets in ES (**Figure 5B**). We then chose the commonly activated or repressed gene sets between the two and eventually obtained 5 activated gene sets, namely DNA repair, E2F targets, G2M checkpoint, and MYC targets V1/2, (**Figures 5C, E**), as well as 4 repressed gene sets, namely epithelial mesenchymal transition, myogenesis, TNFA signaling *via* NFKB, and UV response DN (**Figures 5D, F**).

### Subcellular Localization of FOXP4-AS1

**Figure 6:**
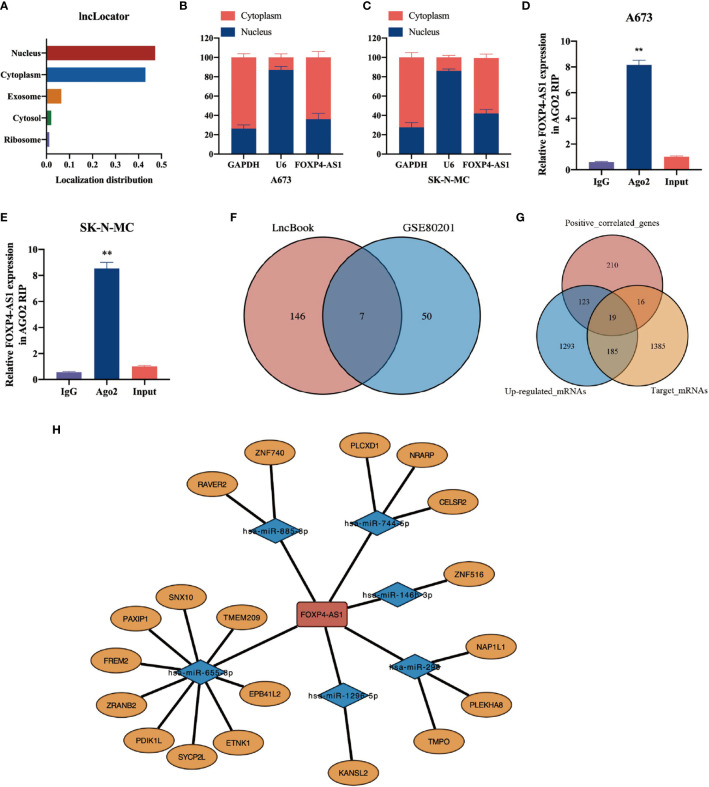
Construction of a ceRNA modulatory network of FOXP4-AS1 in ES. **(A)** The subcellular localization of FOXP4-AS1 was predicted by the lncLocator tool. **(B, C)** Subcellular localization of FOXP4-AS1 was analyzed by nuclear and cytoplasmic separation experiments in A673 and SK-N-MC cells. **(D, E)** Ago2-IP experiment revealed that FOXP4-AS1 can bind to Ago2 and participate in the miRNA-mediated mRNA silencing function. Input represents a sample of the solubilized membranes prior to incubation with the antibody-bound beads. **(F)** Prediction of target miRNAs for FOXP4-AS1 using LncBook. The predicted miRNAs were combined with those down-regulated in Ewing sarcoma to reveal seven miRNAs that were finally incorporated into the ceRNA network. **(G)** Target mRNAs of 7 ceRNA-miRNAs were predicted using miRDB and Targetscan databases. Up-regulated mRNAs (logFC> 0.5, adj.p.value <0.05) were obtained through ES microarray data. We incorporated the intersection between the three gene sets (predicted target mRNAs, up-regulated mRNAs, FOXP4-AS1 positively correlated genes) into the ceRNA network, and obtained a total of 19 potential ceRNA-mRNAs. **(H)** Visualization of the ceRNA network using Cytoscape. **p < 0.01.

Previous researches have documented that LncRNA is closely linked to its subcellular localization. To elucidate the underlying mechanism of FOXP4-AS1 action in Ewing sarcoma, we used the lncLocator tool (http://www.csbio.sjtu. edu.cn/bioinf/lncLocator) to predict its subcellular localization, and found that it was present in the cytoplasm, nucleus, and extracellular vesicles (**Figure 6A**). Then, we employed a nuclear and cytoplasmic separation experiment to analyze subcellular localization of FOXP4-AS1 in Ewing sarcoma cell lines A673 and SK-N-MC. Results showed that FOXP4-AS1 was predominantly localized in the cytoplasm of both cell lines (**Figures 6B, C**).

### A ceRNA Regulatory Network of FOXP4-AS1 in ES

We hypothesized that LncRNA in the cytoplasm can affect the expression of downstream mRNAs by adsorbing miRNA. Since FOXP4-AS1 is primarily localized in the cytoplasm of ES cells, we speculated that it may have ceRNA function. Therefore, we performed the Ago2-IP experiment to determine whether FOXP4-AS1 can bind to Ago2 and participate in the miRNA-mediated mRNA silencing. Data illustrated that FOXP4-AS1 was remarkably enriched in Ago2-IP in ES cells (**Figures 6D, E**), suggesting that it plays a role in miRNA-sponging and exerts a ceRNA function. Based on these results, we constructed a ceRNA network of FOXP4-AS1 in ES. Through the LncBook website, we obtained 153 miRNAs with potential binding sites for FOXP4-AS1. Notably, 50 miRNAs were downregulated (logFC < -0.5, p-value < 0.05) in ES based on the GSE80201 dataset. Thereafter, we incorporated the intersection between the two into the ceRNA network, and obtained a total of 7 potential ceRNA-miRNAs. These included miR-885-3p, miR-298, miR-744-5p, miR-1258, miR-1296-5p, miR-146b-3p, and miR-655-3p (**Figure 6F**).

We employed miRDB (http://mirdb.org) and Targetscan (http://www.targetscan.org/vert_71) databases to predict target mRNAs of the 7 ceRNA-miRNAs, and obtained up-regulated mRNAs (logFC > 0.5, adj.p.value <0.05) through ES microarray data (GSE70826, GSE17618, GSE48022, GSE90970, GSE70826). Thereafter, we incorporated the intersection between the three gene sets (predicted target mRNAs, up-regulated mRNAs, FOXP4-AS1 positively correlated genes) into the ceRNA network, and obtained a total of 19 potential ceRNA-mRNAs (**Figure 6G**). Finally, we constructed a ceRNA network comprising FOXP4-AS1, 7 miRNAs, and 19 mRNAs (**Figure 6H**), which revealed the prospective mechanism of FOXP4-AS1 action.

### GSEA Screens the Molecular Mechanism of FOXP4-AS1

**Figure 7:**
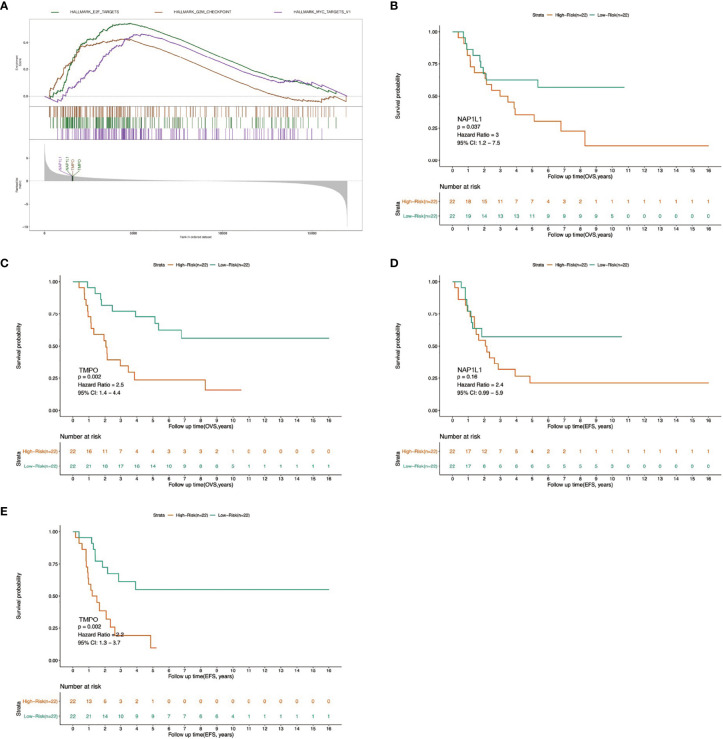
Determination of the molecular mechanism of FOXP4-AS1 by GSEA analysis. **(A)** Across GSVA and GSEA datasets, two crucial genes associated with commonly up-regulated cascades were identified in the ceRNA network. **(B–E)** Survival analysis and univariate Cox regression analysis revealed association of the two crucial genes with overall survival (OVS) and event-free survival (EFS) of ES patients.

To elucidate the potential molecular mechanism of FOXP4-AS1 action, the mRNAs of the ceRNA network were overlapped with gene sets from previous GSEA results. We found that two genes were the crucial genes of the remarkably enriched Hallmark gene sets. Specifically, NAP1L1 is the core gene of MYC TARGETS V1, E2F TARGETS gene set, whereas TMPO is the core gene of G2M CHECKPOINT, E2F TARGETS gene set (**Figure 7A**). Besides, overall survival analysis depicted that elevated expression of NAP1L1 and TMPO in ES patients was predicted dismal prognosis (p <0.05, **Figures 7B, C**), while event-free survival analysis illustrated that only TMPO was correlated with poor prognosis of individuals with ES (p<0.05, **Figures 7D, E**). Therefore, we speculated that FOXP4-AS1 may affect TMPO expression by sponging miR-298, thereby regulating the malignant phenotype of Ewing sarcoma.

### Correlation Between FOXP4-AS1 Expression and Immune Infiltration

**Figure 8:**
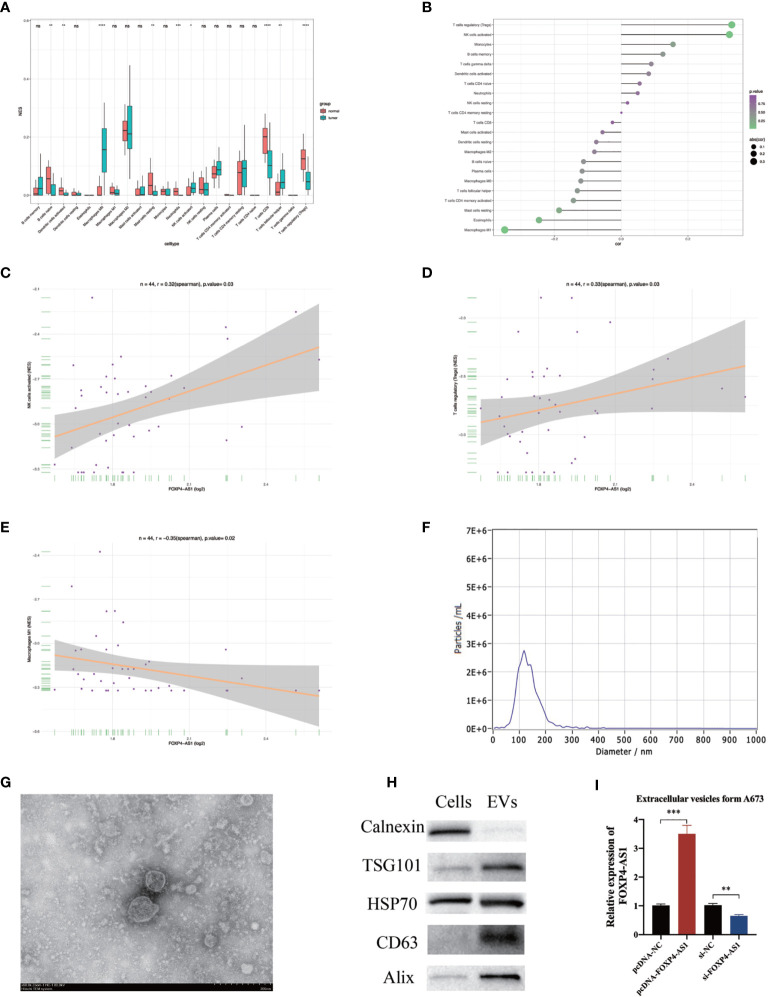
The correlation between FOXP4-AS1 expression and immune infiltration. **(A)** Infiltration level of 22 immune cells in skeletal muscle and ES patient samples was estimated by CIBERSORT. **(B)** The correlation between FOXP4-AS1 expression and immune cell enrichment level was analyzed by Spearman correlation analysis. **(C, D)** FOXP4-AS1 expression was remarkably positively correlated with T cells regulatory (Tregs), NK cells activated. **(E)** FOXP4-AS1 expression was remarkably negatively correlated with Macrophages M1. **(F)** The particle size of the extracellular vesicles was analyzed with Nanoparticle Tracking Analysis (NTA). **(G)** The morphology of extracellular vesicles was determined by Transmission electron microscopy (TEM). **(H)** Western blot was used to quantify the protein levels of markers of extracellular vesicles. **(I)** FOXP4-AS1 expression in A673-derived extracellular vesicles following overexpression or knockdown of FOXP4-AS1 in A673 cells. *p < 0.05, ^**^p < 0.01, ^***^p < 0.001, ****p < 0.0001, ns, non-significant.

Previous investigations have illustrated that invading immune cells have prognostic value in many human tumors (31, 32). Therefore, we estimated infiltration levels of 22 immune cells between skeletal muscle and ES patients (GSE17674). Results showed that ES tissues generally contained higher proportions of M0 macrophages, activated NK cells, and T follicular helper cells, but relatively lower fractions of naive B cells, activated Dendritic cells, resting Mast cells, Neutrophils, CD8 T cells, and T regulatory (Tregs) cells, compared to skeletal muscles (**Figure 8A**). Spearman correlation in ES patients (GSE17618) revealed that FOXP4-AS1 was remarkably associated with immune cell enrichment of three immune cells, namely Tregs, activated NK cells, and Macrophages M1 (**Figures 8B–E**). Overall, these results indicated that atypical expression of FOXP4-AS1 might be playing a crucial role in the tumor immune microenvironment.

### Patterns of FOXP4-AS1 Expression in Extracellular Vesicles

As mentioned above, impaired FOXP4-AS1 expression alters immune cell invasion in the tumor microenvironment, while transfer of RNA from tumor cells to the tumor immune microenvironment mediated by extracellular vesicles may disrupt immune cell infiltration and promote tumor progression. Therefore, we analyzed patterns of FOXP4-AS1 expression in extracellular vesicles derived from ES cells A673. NTA, TEM, and western blots were used to validate the extracellular vesicles isolation and purification. NTA illustrated that the average size of extracellular vesicles was 135 nm for all participants (**Figure 8F**). TEM verified extracellular vesicles morphology and size(**Figure 8G**). Western blot proved the presence of extracellular vesicles markers, including HSP70, TSG-101, Alix, and CD63 (33) (**Figure 8H**). Calnexin was seen as a cellular marker indicating that cellular contamination was not detected in the EVs components (34). qRT-PCR revealed that overexpression of FOXP4-AS1 in ES cells resulted in its upregulation it in extracellular vesicles. In contrast, knocking down FOXP4-AS1 in ES cells downregulation this expression in extracellular vesicles (**Figure 8I**). These results indicated that FOXP4-AS1 may modulates the ES immune microenvironment in an extracellular vesicle-mediated manner.

## Discussion

Numerous researches have documented that impaired lncRNA expression plays an indispensable role in tumorigenesis and development (35). In fact, several lncRNAs are up-regulated in tumors, where they reportedly promote tumor progression (36–38). Some researches have also documented that FOXP4-AS1 is upregulated in various tumors, with this phenomenon confirmed to be a poor prognostic factor for tumors (39–42). The findings herein suggested that FOXP4-AS1 was upregulated in a variety of tumor samples from the TCGA dataset. Particularly, FOXP4-AS1 was highly expressed in ES tissues, with patients overexpressing this gene also found to have poor prognosis. This suggested that FOXP4-AS1 may be regulating tumorigenesis and development of Ewing sarcoma.

Previous investigations have documented that tumor-related lncRNA can modulate tumor growth, migration, invasion, apoptosis, and cell cycle, as well as other biological processes (43–46). In the present study, we used CCK-8, transwell migration and invasion experiments to reveal FOXP4-AS1’s role in Ewing sarcoma. Specifically, downregulating this gene repressed growth, migration, and invasion of ES cells, whereas its overexpression significantly improved these processes. Overall, these data show that FOXP4-AS1 acts as a tumor-promoting factor during progression of ES.

To elucidate the underlying mechanism of FOXP4-AS1 action in Ewing sarcoma, we used a Spearman correlation, and GO enrichment of Hallmark Gene Sets analyses and constructed a PPI network to screen for significantly correlated factors. Our results revealed that FOXP4-AS1 was associated with various functions, including post-translational protein modification, endoplasmic reticulum lumen, Sin3 complex, condensed chromosome kinetochore, centromeric region, chromosome, centromeric region, DNA repair, condensed chromosome, interstrand cross-link repair and double-strand break repair, among others. The hallmark gene set enrichment analysis showed that FOXP4-AS1 was further associated with various signaling cascades, consisting of E2F targets, G2M checkpoint, MYC targets V1, epithelial mesenchymal transition, p53 cascade, glycolysis, mtorc1 signaling, DNA repair, and protein secretion. Taken together, these data indicate that FOXP4-AS1 participates in Ewing sarcoma development and occurrence by regulating several signaling cascades.

Previous studies have shown that lncRNAs have different molecular mechanisms based on different subcellular localization (47, 48). In Ewing sarcoma, the present study found that FOXP4-AS1 is predominantly localized in the cytoplasm, where the ceRNA mechanism is one of the main mechanisms of lncRNA action. Therefore, we constructed a ceRNA network, comprising FOXP4-AS1, 7 miRNAs, and 19 mRNAs, to reveal the potential molecular mechanism of FOXP4-AS1 action. To further clarify this molecular mechanism, the mRNAs of the ceRNA network were overlapped with gene sets from previous GSEA results. We found that FOXP4-AS1 may regulate the expression of TMPO by sponging miR-298, thereby regulating the malignant phenotype of Ewing sarcoma.

The TME (tumor microenvironment) refers to the surrounding microenvironment of tumor cells, including the surrounding bone marrow-derived inflammatory cells, vascular immune cells, fibroblasts, extracellular matrix, various cytokines, and chemokines (32, 49, 50). Previous studies have shown that immune cells in the TME play an essential role in occurrence, development, and metastasis of tumors (32, 51). On the other hand, lncRNAs have been shown to affect levels of immune cell invasion in the TME, thereby affecting tumorigenesis and tumor development (52, 53). Results of the present study revealed that three immune cells, namely Tregs, activated NK cells, and M1 macrophages, were significantly associated with FOXP4-AS1, indicating that atypical expression of FOXP4-AS1 might be playing a crucial role in the tumor immune microenvironment.

Extracellular vesicles (EVs) act as essential communication tools between tumor cells and the tumor microenvironment (54, 55). Specifically, tumor-derived extracellular vesicles can deliver lncRNAs to regulate levels of immune cell infiltration and associated with the diagnosis and prognosis of tumor (56, 57). Our results showed that FOXP4-AS1 could be delivered by ES cell-derived extracellular vesicles and may affect the levels of immune cell infiltration in the TME. It indicates that ES cell-derived extracellular vesicles may affect the composition of the tumor microenvironment by transmitting FOXP4-AS1. However, the function, diagnostic or prognostic values of exosomal FOXP4-AS1 still need further investigation in ES.

## Conclusion

Taken together, our findings revealed that FOXP4-AS1 is a poor prognostic factor of Ewing sarcoma, and plays a crucial role in enhancing cell proliferation, migration, as well as invasion. Functionally, FOXP4-AS1 may regulate the malignant phenotype of Ewing sarcoma by upregulating TMPO by sponging miR-298. ES cell-derived extracellular vesicles may affect the infiltration level of immune cells in TME by transmitting FOXP4-AS1.

## Data Availability Statement

The raw data supporting the conclusions of this article will be made available by the authors, without undue reservation.

## Ethics Statement

The studies involving human participants were reviewed and approved by The Second Affiliated Hospital of Nanchang University.

## Author Contributions

JX and LW performed the experiments and generated data. JX, LH, CW, ZL, WD, SM, ZZ, and HY analyzed data. JX, LW, and KC designed the experiments. JX and KC wrote the manuscript. All authors contributed to the article and approved the submitted version.

## Funding

This study was supported by the National Natural Science Foundation of China (No. 81860473), Key project of Natural Science Foundation of Jiangxi Provincial (No. 20202ACB206004), Major Discipline Academic and Technical Leaders Training Program of Jiangxi Province (No. 20204BCJ22026).

## Conflict of Interest

The authors declare that the research was conducted in the absence of any commercial or financial relationships that could be construed as a potential conflict of interest.

## Publisher’s Note

All claims expressed in this article are solely those of the authors and do not necessarily represent those of their affiliated organizations, or those of the publisher, the editors and the reviewers. Any product that may be evaluated in this article, or claim that may be made by its manufacturer, is not guaranteed or endorsed by the publisher.
