# Supplementary material for: LncRNA FOXP4-AS1 Promotes Progression of Ewing Sarcoma and Is Associated With Immune Infiltrates
Source: Front Oncol. 2021 Oct 26;11:718876. doi: 10.3389/fonc.2021.718876 (PMC8577041; doi:10.3389/fonc.2021.718876)
Supplement: Supplementary file 1 [file DataSheet_1.docx]

| GEO data sets | Platform | Samples |
| --- | --- | --- |
| GSE17618 | GPL570(Affymetrix Human Genome U133 Plus 2.0 Array) | 44 ES patients and 11 ES cell lines |
| GSE17674 | GPL570(Affymetrix Human Genome U133 Plus 2.0 Array) | 44 ES patients and 18 normal skeletal muscle |
| GSE48022 | GPL570(Affymetrix Human Genome U133 Plus 2.0 Array) | 4 BM-MSCs |
| GSE90970 | GPL570(Affymetrix Human Genome U133 Plus 2.0 Array) | 3 BM-MSCs |
| GSE70826 | GPL570(Affymetrix Human Genome U133 Plus 2.0 Array) | 5 ES cell lines and 1 MSCs |
| GSE80201 | GPL17603 (miRXplore TM Microarray) | 20 ES patients and 4 MSCs |

Table S1. The microarray data sets obtained from GEO database. Abbreviations: MSCs, mesenchymal stem cells.

| siRNA | Primer sequence（5′ to 3′） |
| --- | --- |
| FOXP4-AS1-si-1-Foward | CAGAGUUUAUUGUUAUCAA |
| FOXP4-AS1-si-1-Reverse | UUGAUAACAAUAAACUCUG |
| FOXP4-AS1-si-2-Foward | GGUCUGCUGAAGAUGUCAA |
| FOXP4-AS1-si-2-Reverse | UUGACAUCUUCAGCAGACC |
| FOXP4-AS1-si-3-Foward | GGGUGAAGAUGCUGAAGUU |
| FOXP4-AS1-si-3-Reverse | ACGUGACACGUUCGGAGAA |
| si-NC-Foward | UUCUCCGAACGUGUCACGU |
| si-NC-Reverse | ACGUGACACGUUCGGAGAA |

Table S2. Sequences of small interfering (si)RNA.

| Primer | Primer sequence（5′ to 3′） |
| --- | --- |
| FOXP4-AS1-Foward | GTGAGCTTCTGGGTTCGACA |
| FOXP4-AS1-Reverse | ATTGAGGGTTAGGGCAGCAC |
| GAPDH-Foward | GAGTCAACGGATTTGGTCGT |
| GAPDH-Reverse | TTGATTTTGGAGGGATCTCG |
| U6-Foward | CTCGCTTCGGCAGCACA |
| U6-Reverse | AACGCTTCACGAATTTGCGT |

Table S3. Primer sequence.
